# An Improved Software Source Code Vulnerability Detection Method: Combination of Multi-Feature Screening and Integrated Sampling Model

**DOI:** 10.3390/s25061816

**Published:** 2025-03-14

**Authors:** Xin He, Asiya, Daoqi Han, Shuncheng Zhou, Xueliang Fu, Honghui Li

**Affiliations:** College of Computer and Information Engineering, Inner Mongolia Agricultural University, Hohhot 010018, China; hx_121382024@163.com (X.H.); asiya@imau.edu.cn (A.); 15623587331@163.com (D.H.); zhousc1121@163.com (S.Z.); fuxl@imau.edu.cn (X.F.)

**Keywords:** source code vulnerability detection, abstract syntax tree (AST), multi-feature screening, integrated oversampling, Bi-LSTM

## Abstract

Vulnerability detection in software source code is crucial in ensuring software security. Existing models face challenges with dataset class imbalance and long training times. To address these issues, this paper introduces a multi-feature screening and integrated sampling model (MFISM) to enhance vulnerability detection efficiency and accuracy. The key innovations include (i) utilizing abstract syntax tree (AST) representation of source code to extract potential vulnerability-related features through multiple feature screening techniques; (ii) conducting analysis of variance (ANOVA) and evaluating feature selection techniques to identify representative and discriminative features; (iii) addressing class imbalance by applying an integrated over-sampling strategy to create synthetic samples from vulnerable code to expand the minority class sample size; (iv) employing outlier detection technology to filter out abnormal synthetic samples, ensuring high-quality synthesized samples. The model employs a bidirectional long short-term memory network (Bi-LSTM) to accurately identify vulnerabilities in the source code. Experimental results demonstrate that MFISM improves the F1 score performance by approximately 10% compared to existing DeepBalance methods and reduces the training time to 2–3 h. These results confirm the effectiveness and superiority of MFISM in source code vulnerability detection tasks.

## 1. Introduction

With the popularization of digital services and software products in daily life, ensuring software security has become a crucial issue [1]. Furthermore, software vulnerability detection technology has become a highly concerned issue in the software industry. In the field of software security, “vulnerabilities” refer to defects or omissions in software that attackers can exploit to damage the system. Existing research indicates that the number of vulnerabilities is positively correlated with the amount of software development, which further exacerbates people’s concerns about software security, especially software vulnerabilities. According to statistics, by 2020, the number of security vulnerabilities disclosed by the National Vulnerability Database (NVD) in the United States had reached 18,325 [2]. As of 10 October 2022, there were 186,011 entries in the Common Vulnerabilities and Exposures (CVE) dataset, and the number of vulnerabilities showed an explosive growth trend. Recent studies have shown that as more digital services and products are developed, the number of software vulnerabilities will continue to increase in the coming years [3]. Previous developers mainly relied on rule-based code analysis and symbolic execution techniques [4]. Although these methods can effectively detect vulnerabilities, they can generate high false-positive rates, require a lot of manual verification, and instead lead to increased workload. Therefore, in order to improve the efficiency of code vulnerability detection, extensive research has been devoted to using deep learning models for automatic vulnerability detection [5].

In recent years, the rapid development of deep learning (DL) technology has provided a solid technical foundation for software vulnerability detection [6,7,8,9]. Currently, deep learning-based code vulnerability detection methods are mainly divided into two categories: sequence-based methods and graph-based methods. The sequence-based method converts the source code into serialized form, treating the code elements as tokens [10]. Using long short-term memory (LSTM) or convolutional neural network (CNN) [11] models to learn sequence features for vulnerability detection; The graph-based method converts source code into heterogeneous structures such as the abstract syntax tree (AST), control flow graph (CFG), and program dependency graph (PDG) and then uses neural network models to capture local structures and dependency relationships. Compared with code sequences, this can provide richer syntax and structure to detect vulnerabilities [12].

However, software vulnerability detection is facing a serious problem of “class imbalance”, manifested by insufficient training data and uneven sample distribution [13,14]. When training the model, the majority class samples in the dataset are usually much more than the minority class samples, resulting in the final evaluation performance of the model being significantly biased towards the majority class during training. Due to the insufficient sample size of minority classes, the model finds it difficult to learn effective information from them, thereby reducing its ability to recognize minority classes. Even if the overall classification accuracy is high, the performance of minority classes may still be poor [15]. Liu [13] et al. conducted specific research on sample class imbalance and found that the problem of “class imbalance” significantly reduces the accuracy of model recognition of vulnerable samples and also affects the recognition performance of deep learning models on minority class samples.

Lu [14] et al. noted that current vulnerability detection methods based on deep learning face two primary limitations: Firstly, model training times are lengthy. For instance, Li [16] et al. employed the Feature-Attention Graph Convolutional Network (FA-GCN) for vulnerability detection, yet the training process took up to 9 days. Moreover, the size of the training dataset constrained the model’s performance. Pathak [17] and colleagues demonstrated that feature selection techniques can reduce the model training time in malware detection, with negligible accuracy loss. Addressing these issues, this paper introduces a novel approach aimed at tackling the lengthy training cycles and limited training data in software vulnerability detection models. The experimental results indicate that our proposed method not only significantly reduces model training times but also maintains recognition accuracy. Additionally, an enhanced data processing strategy has been implemented, effectively boosting the accuracy of software vulnerability detection. Specifically, this paper employs a feature representation learning approach that integrates machine learning (ML) and deep learning (DL), addressing the challenge of automatically extracting deep vulnerable code features. Compared to shallow features reliant on domain knowledge, these deep features encapsulate more vulnerability-related information.

The method proposed in this article is based on the assumption of Liu et al. [13] that vulnerable programming habits are associated with multiple potential vulnerabilities, which can be identified by analyzing the AST of the code program. To address the issue of class imbalance in the dataset, this paper introduces the integrated oversampling technique (IOS), which can generate high-quality minority class composite samples and combine outlier detection techniques to balance the distribution of vulnerability (minority class) and non-vulnerability (majority class) samples, thereby improving the overall performance of the model.

The main contributions of this study can be summarized as follows:

1. This article proposes a feature selection method that combines machine learning and deep learning. The method extracts source code features based on AST, selects high importance features through analysis of variance (ANOVA), and uses a bidirectional long short-term memory (Bi-LSTM) model for deep learning, effectively solving the problem of the long model training time.
2. We propose a data balancing scheme based on integrated oversampling (IOS), which improves the quality of synthesized samples and solves the problem of class imbalance in the dataset by generating synthetic samples of vulnerability sample classes and combining them with outlier detection.
3. Through extensive experiments, this method has shown good performance in software vulnerability detection in practical projects, with significant improvements in accuracy and efficiency compared to existing methods.

The rest of this article is organized as follows. Section 2 introduces the relevant work in the field of software source code vulnerability detection. Section 3 introduces the MFISM proposed in this article. Section 4 evaluates and analyzes the vulnerability detection model proposed in this article. Section 5 summarizes the work of this article and provides a direction for future research.

## 2. Related Work

As the complexity of software systems continues to rise, software vulnerability detection has emerged as a crucial aspect of network security. Vulnerability detection tasks involve analyzing software systems or source code to identify vulnerabilities. The primary objective is to locate and rectify security vulnerabilities within software, thereby enhancing system security and minimizing the risk of attack.

Traditionally, vulnerability detection relied on experts to perform time-consuming and error-prone manual annotation tasks. However, with the introduction of deep learning technology, this situation is changing. Zheng et al. [18] conducted a comparative evaluation between traditional machine learning methods (such as random forest, gradient boosting decision tree, and support vector machine methods) and deep learning methods (such as CNNs and recurrent neural networks (RNNs)). The results indicated that, in most vulnerability detection tasks, the Bi-LSTM structure in an RNN performs optimally. This demonstrates that deep learning methods based on datasets are superior to traditional machine learning methods in detecting software vulnerabilities. In the following sections, we delve into two prominent categories of deep learning-based vulnerability detection methods: graph-based methods and sequence-based methods. These approaches leverage the structural and sequential properties of source code to enhance detection capabilities, each with its own strengths and challenges.

### 2.1. Graph-Based Vulnerability Detection Method

Graph-based vulnerability detection methods have gained traction due to their ability to capture structural information from source code, such as abstract syntax trees (ASTs), control flow graphs (CFGs), and data flow graphs (DFGs). These methods excel by leveraging graph neural networks (GNNs) and other deep learning techniques. For instance, BGNN4VD [6] integrates AST, CFG, and DFG to extract syntax and semantic information, using bidirectional GNNs and CNNs for feature learning and vulnerability detection. While it improves precision and accuracy, its high complexity results in long training and inference times. Similarly, LineVD [19] treats statement-level vulnerability detection as a node classification task, combining GNNs and Transformers to manage code dependencies. It performs well in pinpointing vulnerabilities but struggles with control structures and has a high computational overhead.

Other methods, such as ReGVD [20], use GCNs or GGNNs for vulnerability detection, achieving state-of-the-art performance. Although simple to implement and effective in capturing local code structures, it is limited to function-level detection and struggles with complex code. TI-MVD [21], on the other hand, constructs a temporal heterogeneous graph to capture malware evolution patterns, excelling in terms of runtime efficiency for large-scale API interactions, but it is limited to malware detection. VulGAI [22] generates images from code graphs and uses CNNs for efficient and precise detection, performing well on real-world datasets but potentially losing some semantic information and being limited to C/C++ code.

Advanced models like VulTR [23] employ multi-layer key feature enhancement, using GNNs to process program dependency graphs (PDGs) and improve feature extraction. However, they suffer from high complexity and computational overheads. Similarly, CEVulDet [24] removes unimportant nodes from PDGs using centrality analysis and employs CNNs for detection, enhancing recall rates but remaining limited to C/C++ code and incurring high computational costs.

In summary, graph-based vulnerability detection methods significantly improve precision and accuracy by leveraging the structural information of the source code. However, challenges such as high model complexity, limited generalization, and computational inefficiency remain unresolved. These methods represent a promising direction but require further optimization for broader applicability.

### 2.2. Sequence-Based Vulnerability Detection Method

Sequence-based vulnerability detection methods have gained significant attention in recent years, leveraging natural language processing (NLP) techniques to treat source code as text sequences. These methods convert code into text representations, such as word vectors or character vectors, and utilize deep learning models like recurrent neural networks (RNNs), convolutional neural networks (CNNs), and Transformers for feature extraction and classification.

For instance, Li et al. [25] proposed VulDeePecker, a system that employs Bi-LSTM networks to analyze code snippets. This approach effectively reduces the reliance on manual feature engineering by converting code snippets into vector representations. However, VulDeePecker may struggle with complex code structures, where graph-based methods tend to perform better.

Building on this foundation, Zhen Li et al. introduced the SySeVR [26] framework, which enhances vulnerability detection by extracting syntactic and semantic vulnerability candidate codes and applying deep neural networks. SySeVR’s use of Bidirectional Gated Recurrent Units (BGRUs) significantly improves detection performance across various vulnerability types. Despite its strengths in integrating syntax and semantics, SySeVR’s syntactic feature coverage is limited, potentially missing some vulnerability patterns.

Another notable advancement is VulBERTa [27], proposed by Hanif et al., which simplifies source code pretraining using the RoBERTa model and fine-tuning with Multi-Layer Perceptrons (MLPs) or CNNs. VulBERTa captures code syntax and semantics effectively, outperforming baseline methods on multiple datasets. Its simplicity and scalability make it suitable for various programming languages. However, the pretraining process is computationally expensive, and its detection capability for emerging 0-day vulnerabilities is limited.

Complementing these efforts, Zhangyin Feng et al. [28] introduced CodeBERT, a Transformer-based pretraining model specifically designed for source code and natural language processing. Pretrained on large-scale code and text data, CodeBERT captures deep semantic information, performing well in tasks like code searching and generation. While CodeBERT’s semantic capture is effective and applicable to multiple programming languages, the pretraining process is resource-intensive, and specific vulnerability detection tasks may require further fine-tuning.

To further enhance detection performance, Wenxuan Li et al. [29] proposed the COCL method, a deep learning-based framework that incorporates contrastive and curriculum learning. COCL improves model generalization and detection performance, effectively handling complex vulnerabilities and boosting recall rates. However, the introduction of these advanced learning techniques increases training complexity and cost, especially for large datasets.

Despite significant progress in performance, sequence-based methods still face challenges such as high model complexity and limited generalization capabilities. Future work should focus on improving model efficiency and scalability while addressing these limitations to enhance the applicability of these methods to a broader range of programming languages and vulnerability types.

### 2.3. Applications and Challenges of Deep Learning in Software Vulnerability Detection

Ban et al. [30] noted through experiments that class imbalance significantly negatively impacts the performance of software vulnerability detection. Additionally, Johnson et al. [31] highlighted that in the field of deep learning, there are relatively few solutions for addressing unbalanced data. Therefore, the issue of class imbalance must be carefully considered when utilizing deep learning methods for vulnerability detection. Simultaneously, other challenges, such as excessively long training times and insufficiently sized trainable datasets, also need to be addressed.

In software vulnerability detection, the class imbalance problem is a critical challenge. This issue stems from the fact that the number of minority class samples (such as vulnerability samples) is significantly lower than that of majority class samples (such as normal code samples). This imbalance causes models to bias toward the majority class during training, thereby reducing their ability to identify minority class samples. The imbalance not only affects the model’s generalization capability but also masks the poor performance of the minority class, even if the overall classification accuracy is high [15]. Liu [13] et al. conducted an in-depth study of the class imbalance problem and found that it significantly reduces the model’s recognition accuracy for minority class samples. To address this issue, they proposed a class rebalancing approach based on fuzz testing. By generating synthetic samples using fuzz testing techniques, the number of minority class samples can be effectively increased, thereby improving the model’s training effectiveness. This method not only helps to mitigate the problem of class imbalance but also enhances the model’s ability to identify minority class samples.

Despite the development of various methods to address the class imbalance problem, several challenges remain. For example, the complexity of data distribution, the extreme imbalance in sample quantities, and the tendency of models to overfit to the minority class continue to restrict model performance in practical applications. Additionally, the long training times of deep learning models are a significant issue, especially when dealing with large-scale datasets or complex models. Excessive training times can substantially increase computational costs and reduce model usability. These challenges highlight the necessity of developing more robust and adaptive solutions.

## 3. Research Methods

**Figure 1:**
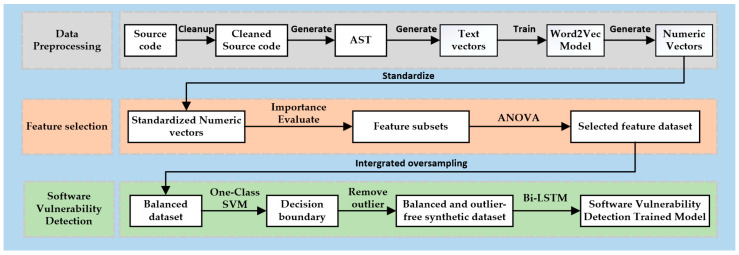
The structural framework of the MFISM.

This article proposes a source code vulnerability detection method called MFISM, which combines machine learning and deep learning techniques. MFISM also focuses on solving the problem of class imbalance in datasets and improves detection accuracy and efficiency through optimized feature selection schemes and integrated oversampling methods. The experiment shows that this method can not only effectively identify potential vulnerabilities in the source code but also cope with the challenges brought about by imbalanced categories in the dataset. Figure 1 illustrates the structural framework of the method proposed in this paper.

The content of this section is organized as follows:

Section 3.1 describes the data preprocessing stage and the feature selection process.

Section 3.2 presents the integrated sampling method, which is used to balance the dataset.

Section 3.3 elaborates on the software source code vulnerability detection approach based on the Bi-LSTM model.

### 3.1. Data Preprocessing and Feature Selection

**Figure 2:**
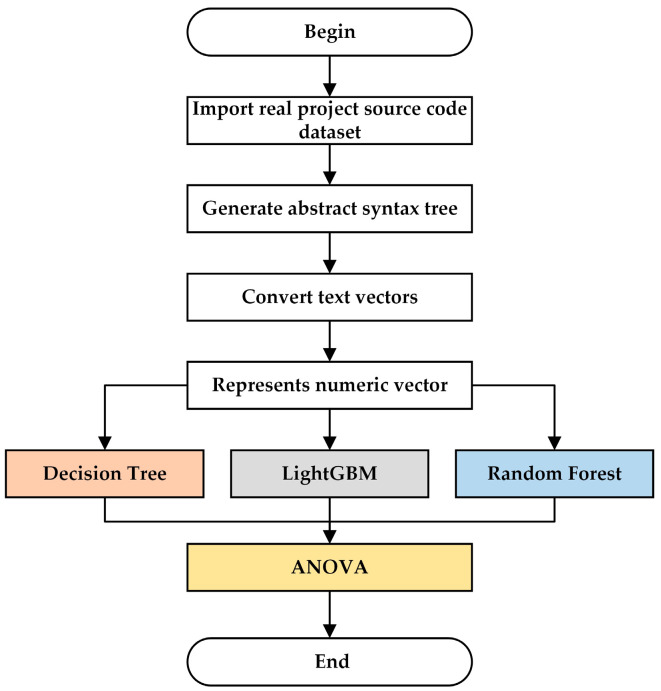
Data preprocessing and feature selection.

The dataset utilized in this experiment is sourced from commonly used datasets in real-world projects, focusing solely on function-level vulnerabilities. Additionally, vulnerabilities spanning multiple functions or files were not considered in this study. The primary processes involved in data preprocessing and feature selection are illustrated in Figure 2.

#### 3.1.1. Data Processing

**Figure 3:**
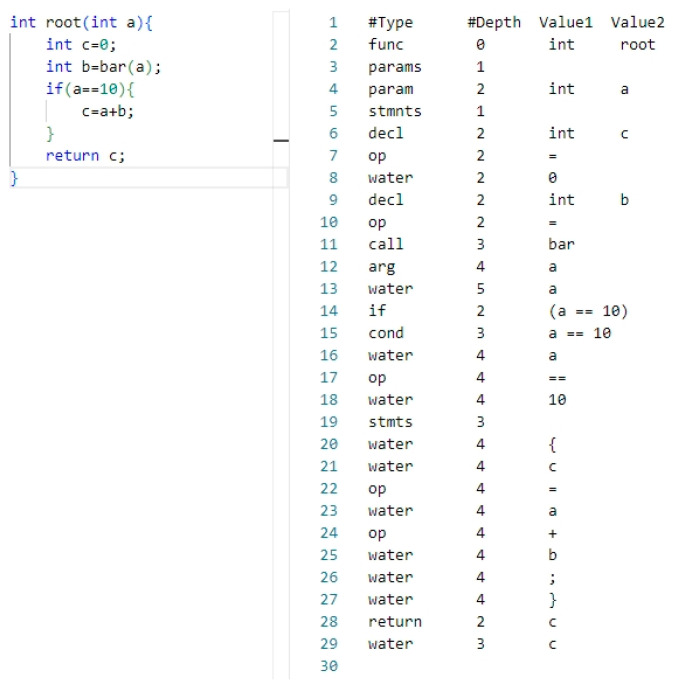
The corresponding AST generated by CodeSensor has the instance source code on the left and its corresponding AST serialization on the right.

When handling software source code, the initial step involves removing non-essential content, such as comments and header files. Following this, the source code is converted into its corresponding AST representation. The AST represents the code’s semantics and structure in a tree-like format, preserving programming patterns. It encompasses all code components within a function, including the function-level control flow. Hence, the AST serves as the program code representation for feature extraction. Nevertheless, parsing the AST within source code poses a challenge, as code compilation typically necessitates a compilation environment. To address this, this article employs CodeSensor for this task. CodeSensor [32], a robust parser rooted in the concept of island grammar [33], excels at extracting AST from source code functions, eliminating the need for supporting libraries and dependencies. With CodeSensor, generating the AST for source code becomes effortless. Specifically, CodeSensor takes the program’s source code function as the input and outputs its AST, presented in a serialized format. This serialized AST format displays the tree structure in tabular format, clearly illustrating the code’s hierarchical structure and logical relationships, as exemplified in Figure 3.

The depth column marks the position of each component within the source code function. For instance, in the second row of the table (located in the right half of Figure 3), the type name “func” and a depth of 0 signify that the function named “root” with a return value of “int” represents the root of the tree. The third row labeled “params” indicates that the function accepts at least one parameter. A depth of 1 denotes that it is the first child node of the root node, and so on. By parsing the AST, we convert it into a vector form while retaining its structural information.

In an AST, the “water” node serves as a placeholder for elements in the source code that do not directly impact the program’s logic. These elements include whitespace characters, comments, and delimiters. When parsing source code, parsers often regard such elements as “moisture”, meaning they have no direct influence on the program’s logic. Consequently, these elements are represented by “water” nodes to act as placeholders. Although these elements exist in the source code, they do not affect the program’s execution logic during syntax analysis. The use of “water” nodes ensures that the original code structure and style are preserved during operations such as code formatting and refactoring.

By utilizing the depth-first traversal (DFT) method to map the nodes of the AST to elements in a vector, each node is consequently mapped to an element of the vector. The sequence of elements within the transformed vector mirrors the hierarchical structure of nodes within the AST. Taking the converted text sequence [root, int, params, param, int, b, stmnts, decl, int, c…] as an example, the root node in the AST is mapped to the first element of the vector, representing the function name. The second element is int, indicating the return type of the root function. The third element represents the parameters of the root function. The fourth and fifth elements are the parameter “param” and its corresponding type, int. These elements collectively define the functionality of the root function, and each element in the vector, along with its order, collectively determines the semantics of the statement. In this way, the hierarchical information of the code structure is preserved, and the AST is represented as a text vector, facilitating subsequent further processing.

Next, we convert the text vector into the corresponding numeric vector. For this process, we employ the Word2Vec [34] model, which can fully consider the semantic structure of code and embed the semantics of each code element. The Word2Vec model was trained using the Continuous Bag of Words (CBOW) architecture. The training process is unsupervised; thus, no labeled data are required. The Word2Vec model is trained on the code bases of three real open-source projects. The training result is a dictionary that maps each code element to its corresponding 100-dimensional vector embedding. Each text element in the vector is mapped to an integer corresponding to it. For instance, if the serial number of types ‘int’ is ‘3’, then it will be mapped to ‘3’, and if the serial number of type ‘void’ is ‘4’, then it will be mapped to ‘4’, and so on. However, the varying lengths of each function code segment led to differences in vector dimensions. To prevent issues arising from abnormal vector dimensions during the subsequent training phase, this paper standardizes the vectors.

**Figure 4:**
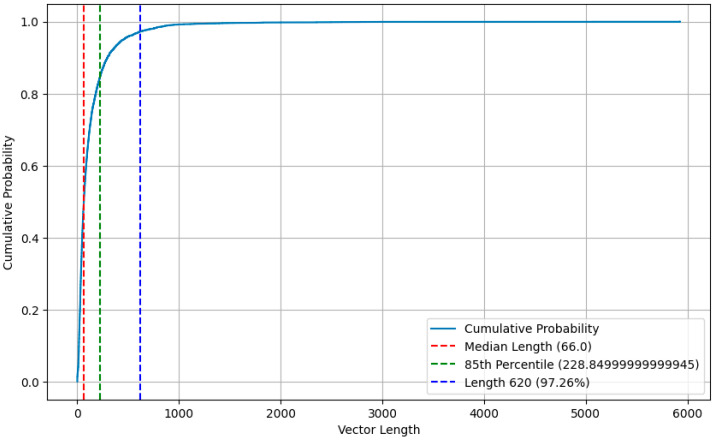
Cumulative probability distribution of vector lengths.

Considering the balance between the length of the vector and the over sparsity, we performed an in-depth analysis of the cumulative probability distribution of the vector length to determine a suitable fill/truncation threshold that strikes a balance between preserving data integrity and processing efficiency. Figure 4 illustrates the cumulative probability distribution for the length of the vector, from which we chose 620 as the threshold. Statistical analysis shows that this threshold is able to cover up to 97.26% of the data points, which means that truncating the vector length to 620 will lead to the loss of little important information, as the vast majority of data points do not exceed this value in the vector length.

With the increase in vector length, the growth trend of cumulative probability gradually flattens, which indicates that the distribution of longer vectors in the dataset is relatively sparse. Choosing 620 as the threshold significantly reduces the complexity of processing sparse data while retaining most of the data. In addition, shorter vector lengths help improve computational efficiency because they take up less storage space and computing resources. Therefore, by truncating the vector length to 620, we were able to optimize the computational performance without sacrificing data integrity.

Further statistical analysis showed that the 5% quantile (228.85) was much smaller than 620, indicating that 620 had significantly exceeded the vector length of most data points. From a statistical point of view, 620 is a reasonable choice because it not only retains the main characteristics of the data distribution but also avoids the possible effects of extreme values. Therefore, the choice of 620 as a threshold is a scientific decision based on data distribution characteristics and statistical analysis, aiming to achieve the best balance between data integrity and computational efficiency. As a result, vectors with more than 620-bit elements will be truncated, while vectors below 620-bit elements will be filled with 0 to reach 620.

During the numerical vector conversion process, serial numbers with leading zeros are skipped to maintain consistency in numerical vector dimensions.

The specific steps for data processing in this section are shown in Algorithm 1.
**Algorithm 1.** Preprocess Source code into Standardized Numeric vector**Input:** Source code**Output:** Standardized Numeric vectors**Step 1: Source code cleanup**   Removing comments, unnecessary header files, etc.;   Cleaned source code; **Step 2: Generate AST**   Use CodeSensor to parse the cleaned source code into an AST. **Step 3: Generate Text vectors**   Initialize empty text vectors, and execute DFT on AST to generate text vectors;**Step 4: Train the Word2Vec model**   Initialize and train the Word2Vec model using text vectors;**Step 5: Generate Numeric vectors**   Initialize an empty Numeric vectors;   Traverse each element in Text vectors:   If the element exists in the Word2vec_model:   Add the vector corresponding to the element to Numeric vectors;   Else:   Add the zero vector to Numeric vectors; **Step 6: Standardizing the Numeric vectors**:   If (Numeric vectors) > LEN:   Truncate Numeric vectors to LEN;   Else:   Pad the Numeric vectors with zero to LEN;

#### 3.1.2. Feature Selection

This article introduces a method that integrates feature selection and feature screening. Initially, multiple machine learning models are employed for feature selection, followed by ANOVA for final feature screening, ensuring the selection of the most representative and discriminative features.

The experiment conducted by Han et al. [35] effectively demonstrates that selecting features based on their importance in machine learning is highly effective and can identify the most representative and discriminative features. In this article, we utilize the feature importance of three machine learning models, namely, random forest [36], decision tree [37], and LightGBM [38], for feature selection. The methods used to evaluate feature importance vary among these models. Random forest employs Gini Impurity to assess feature importance, which is based on the criteria for selecting features during the splitting of decision tree nodes. The decision tree selects features through information gain, calculated by subtracting the conditional entropy of the feature from the initial entropy of the dataset. LightGBM evaluates feature importance based on the amount of Gini coefficient reduction, calculating the sum of reduced Gini coefficients for each feature across all trees and normalizing it to determine the feature’s importance. It is important to note that these models are used with their default settings to ensure consistency and reproducibility in our experiments.

After feature extraction via the aforementioned three distinct machine learning models, these feature subsets are utilized as input data for ANOVA [39] to further filter out features that are crucial to the model’s predictive performance. ANOVA is a statistical method employed to compare the mean differences among three or more groups of data. It determines whether there are significant differences between different groups by analyzing the variance between and within groups. By treating each feature subset as a set of data and subjecting it to ANOVA analysis, features that significantly contribute to model prediction can be identified, while features that have a minor impact on the results can be eliminated.

Specifically, the feature set selected by each machine learning model is regarded as a group, and the features within the group and their corresponding feature importance scores are treated as observed values. One-way analysis of variance is employed to test whether the differences between groups of features are significant. ANOVA performs hypothesis testing by automatically calculating the F statistic and its corresponding *p*-value. The *p*-value represents the probability of observing the current F statistic or a more extreme value under the null hypothesis (i.e., the means of the groups are equal, meaning that the selected features are identical). Typically, the significance level is set at 0.05. If the *p*-value is less than this threshold, then the null hypothesis is rejected, indicating significant differences between groups, and further feature selection can be conducted; otherwise, it is considered that the differences are not significant and feature selection cannot be performed.

ANOVA not only aids in identifying significant features but also ranks their importance, thereby pinpointing which features are most crucial in code vulnerability detection. By integrating feature importance assessment with ANOVA results, a set of the most representative features can be ultimately selected. This approach is more scientific and effective than manually assigning weights to features before selecting the final ones. This process not only enhances the interpretability and generalization ability of the model but also optimizes the number of features and reduces the training time while maintaining model performance.

This method significantly reduces the model training time through multi-dimensional optimization. Its core lies in reducing feature dimensions, optimizing model complexity, improving training efficiency, and reducing the risk of overfitting through scientific feature selection strategies. Specifically, ANOVA is used to screen features significantly related to the target variable, effectively eliminating redundant or irrelevant features, thus significantly reducing the feature dimensions. This process not only reduces the amount of computation during model training but also avoids the “dimension disaster” caused by high-dimensional data. This “dimension disaster” refers to the exponential growth in the number of parameters that the model needs to handle in high-dimensional space, leading to a low training efficiency. After feature selection, the model structure becomes more concise and the complexity is optimized, significantly reducing the number of parameters that need to be processed during the training process. This not only substantially decreases the training time but also facilitates convergence. Additionally, the filtered feature set is more representative and can support model learning more efficiently, enabling the model to achieve better performance with fewer training iterations, thereby further shortening the training time. Simultaneously, removing irrelevant features enhances the model’s generalization ability, significantly reduces the risk of overfitting, improves the stability and adaptability of the model, and further accelerates the convergence speed of the model. In summary, this method significantly reduces the model training time and enhances the model’s generalization ability and stability by reducing feature dimensions, optimizing the model complexity, improving the training efficiency, and reducing the risk of overfitting. This approach provides strong support for efficient model development in practical applications.

By selecting and filtering features based on the aforementioned characteristics, we can ensure the diversity and comprehensiveness of the chosen features and thus more accurately reflect the characteristics of the dataset. Given that different models exhibit varying sensitivities to features, integrating the feature importance evaluation results from various machine learning models offers a more comprehensive perspective. This aids in better understanding the contribution of each feature to source code vulnerability detection and mitigating the bias that may arise from a single model. Utilizing ANOVA for comprehensive feature selection ensures the applicability and effectiveness of the selected features. This multi-model and multi-method evaluation approach facilitates the selection of features that can comprehensively represent vulnerability characteristics from multiple perspectives, thereby enhancing the effectiveness of node division.

The main process of feature selection and screening in this section is shown in Algorithm 2.
**Algorithm 2.** Feature Selection**Input:** Standardized Numeric vectors**Output:** Selected feature subset**Begin** **Step 1: Get Feature subsets**   Initialize models: Random Forest (RF), Decision Tree (DT), LightGBM (LGBM);   Train model on Standardized Numeric vectors;   If model = RF:   Calculate feature importance based on Gini Impurity;   If model = DT:   Calculate feature importance based on Information Gain;   If model = LGBM:   Calculate feature importance based on Gini Index Reduction;   Store feature importance and evaluation results for each model;   Get Feature subsets; **Step 2: Generate the Selected feature dataset:**   For each feature subset do:   one-way ANOVA;   Calculate F-statistic and *p*-value;   If *p*-value < 0.05 then   Reject original hypothesis; (significant difference between groups)   Select features that are significant in ANOVA and ranked top 30 in feature importance;   Else   Retain original hypothesis; (no significant difference between groups)   End if   End for   Merge the feature importance scores and corresponding features for RF, DT, and LGBM;   Sort features according to the feature importance score;   Get Selected feature dataset;**End**

### 3.2. Balanced Dataset Based on Integrated Sampling

Due to the severe class imbalance issue in the dataset, where there is a significant difference in quantity between the majority class (normal code samples) and the minority class (vulnerable code samples), directly training a deep learning model using this imbalanced dataset can lead to model predictions favoring the majority class samples. In this section, normal code samples are collectively referred to as the majority class, and vulnerable code samples are referred to as the minority class. Due to the small number of minority class samples, even after feature selection, there may still be situations where insufficient information is provided during model training, which can weaken the model’s ability to accurately identify vulnerable code. The core goal of vulnerability detection is to accurately predict vulnerable code function segments (i.e., minority class samples). Related research has shown that the class imbalance issue has a significant negative impact on software vulnerability detection. In experiments at the source code level, when the proportion of vulnerable samples is only 0.33%, the model’s recall rate is less than 14%, and the precision rate is also below 47% [13]. This indicates that if no measures are taken to address the class imbalance issue, the effectiveness of the model in detecting vulnerable code will significantly decrease.

**Figure 5:**
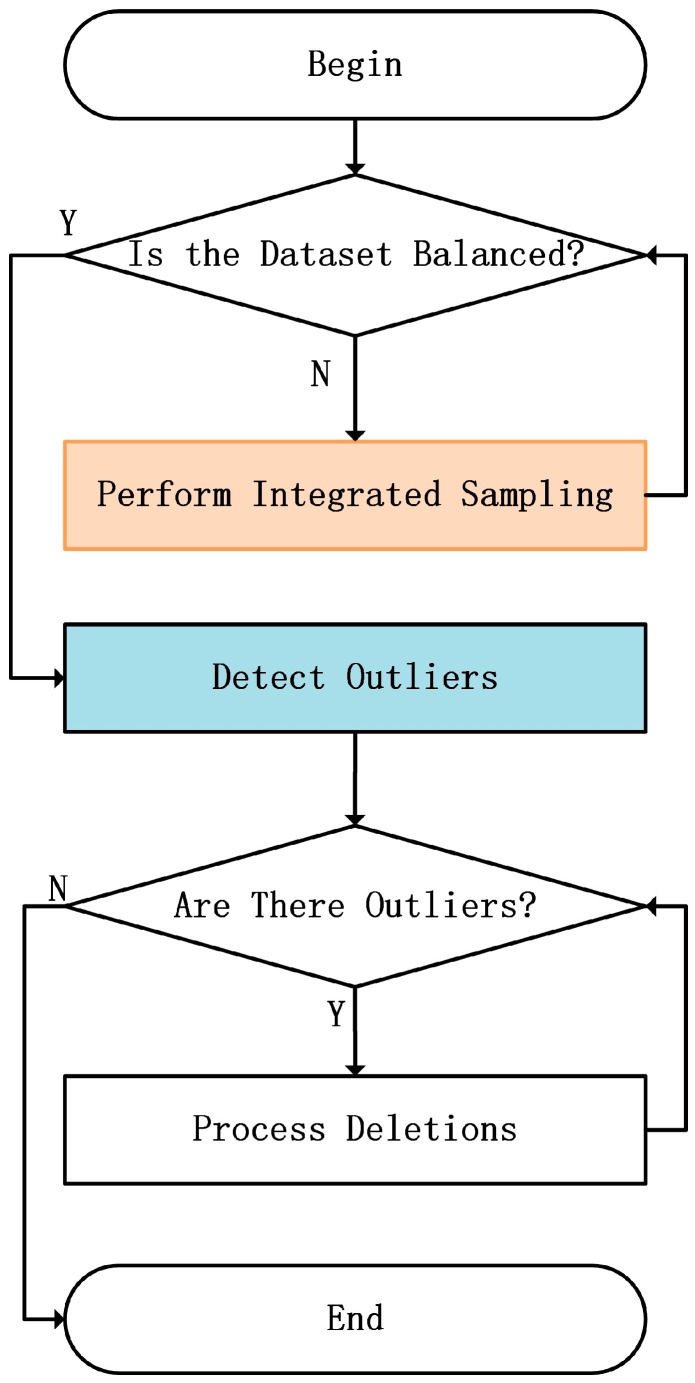
Flowchart based on integrated oversampling technology.

To address the issue of class imbalance, this paper proposes an approach based on the Integration Oversampling Method to tackle the problems present in the dataset. This method initially employs various oversampling techniques to adaptively balance the minority and majority class samples based on the characteristics of sample distribution in the dataset. By generating synthetic samples of minority classes, the number of such samples is increased. Subsequently, outlier detection is performed on the synthetic samples, and any detected outliers are removed. This approach can balance the dataset during the training process and enhance the quality of synthetic samples, thereby improving the model’s ability to identify vulnerable code segments. This method not only increases the number of samples and enhances the overall performance of the model, but also effectively mitigates the prediction bias caused by class imbalance, making the model more accurate in identifying vulnerable code. The flowchart for this part is shown in Figure 5:

This paper adopts four widely recognized integrated oversampling methods: ADASYN [40], SMOTE [41], SVMSMOTE [42], and Borderline-SMOTE [43]. These methods collectively form the core of the data augmentation strategy in this paper. In the process of expanding the original dataset, this study did not adjust or optimize the basic structure of these oversampling techniques but opted for a more direct integration approach. This approach allows each technique to operate independently on the same data sample, generating synthetic minority class samples without mutual interference. By applying these different oversampling methods equally, this paper achieves dataset balance, thereby enhancing the model’s ability to recognize minority class samples without sacrificing data diversity. It is important to note that all these oversampling methods are used with their default parameters to ensure consistency and comparability in the experiments. This integration strategy not only increases the diversity of minority class samples but also helps the model capture more comprehensive feature representations, which is crucial in improving model performance. Additionally, this approach reduces the bias and overfitting risks that may be introduced by a single oversampling method. This multi-technique integration method enables the model to obtain broader sample coverage during data augmentation, thereby enhancing the model’s learning ability for complex data patterns. Comprehensive sample coverage not only enhances the robustness of the model but also improves its generalization ability.

The use of oversampling methods inevitably leads to data noise issues [44]. Since the generated minority samples (synthetic samples) may overlap with the majority samples, resulting in noise, these synthetic samples may mislead model training and affect the reliability of the results. To address this issue, this paper employs outlier detection methods to identify noisy samples that differ from the majority of samples, thereby avoiding their negative impact on model performance. Outliers refer to data points that are significantly different from the majority of samples in the dataset, typically manifesting as points that have a large distance from other data points or do not conform to the expected pattern. In this paper, synthetic samples that have a large distance from the majority of synthetic samples are considered outliers. Therefore, this paper mainly detects and removes outliers through two distance-based detection methods.

The detection process is divided into two parts. Firstly, data points are classified into normal and abnormal points based on the classification algorithm, One-Class SVM [45], and abnormal points are subsequently deleted. Simply phrased, the judgment logic of One-Class SVM is to learn a boundary that maximally retains normal samples and identifies points deviating from this boundary (abnormal points). Then, the local outlier factor (LOF) [46], a density-based method, is used to determine local density. Data points with a lower local density can be identified as abnormal points and deleted. LOF identifies abnormal points by comparing local densities and the density relationships of neighbors, making it a powerful density-based method for detecting abnormal points that are suitable for complex datasets. These two methods capture data anomalies from different perspectives. One-Class SVM focuses on identifying points far from the decision boundary and is suitable for anomaly detection in the absence of labels or incomplete labels, which is also consistent with the situation where synthetic samples overlap with majority samples in oversampling. LOF focuses on deviations in local density. By combining One-Class SVM with boundary conditions and LOF based on density analysis, data noise can be minimized as much as possible.

One-Class SVM obtains a decision boundary through learning from the training dataset. This boundary defines a domain, which is a hypersphere encompassing most of the normal data points. Anomalies are typically located outside this domain. Assuming that anomalies are rare and exhibit significant differences from the distribution of normal data points, the specific formula for this method is as follows:

(1) $$f\left(x\right)=sign\left(\omega^{T}\cdot \phi \left(x\right)+b\right)$$

The optimization problem can be expressed as

(2) $${min}_{\omega ,\xi }\frac{1}{2}{\left\|\omega \right\|}^{2}+\frac{1}{\nu N}\sum_{i=1}^{N}\xi_{i}$$

The constraint conditions are

(3) $$\omega^{T}\phi \left(x_{i}\right)+b\geq 1-\xi_{i},\xi_{i}\geq 0,i=1,2,\ldots N$$

where ω represents the weight vector, $\phi \left(x\right)$ is the mapping function that transforms input data x into a high-dimensional space, b denotes the bias term, the sign function determines whether a data point is normal or abnormal, and ν is a parameter that controls the proportion of abnormal points. During the training process, we first select a kernel function. In this paper, we opt for the RBF (Radial Basis Function) kernel, represented as $Κ\left(x,y\right)=\exp\left(-\Upsilon {\left\|x-y\right\|}^{2}\right)$, where γ serves as the kernel function’s parameter, regulating the distribution of data in the high-dimensional space. Subsequently, we determine the decision boundary by solving an optimization problem.

During the training process, LOF detects outliers by calculating the local outlier frequency (LOF) of data points. LOF computes the k-nearest neighbor distance and reachable distance for each data point and then uses these distances to calculate the local outlier frequency. Data points with a low local outlier frequency, meaning that the density of their neighboring points is significantly lower than that of the data point itself, are considered outliers. The specific process is as follows:

The dataset in this article can be represented as $\{x_{i}{\}}_{i=1}^{N}$, where $x_{i}\in R^{d}$ represents a data sample, d is the feature dimension, and *N* is the number of samples. Firstly, for each data point $x_{i}$, the distance to its k-nearest neighbors is calculated, denoted as $k-dist\left(x_{i}\right)$. Then, the set of its k-nearest neighbors, *N_k_*(*x_i_*), is found, and its local reachable density $lrd\left(x_{i}\right)$ is calculated as follows:

(4) $$lrd\left(x_{i}\right)=\frac{1}{\frac{1}{k}\sum_{x_{j}\in N_{k}\left(x_{i}\right)}dist\left(x_{i},x_{j}\right)}$$

where $dist\left(x_{i},x_{j}\right)$ represents the distance between data points $x_{i}$ and $x_{j}$. Then, for each data point $x_{i}$, its local outlier factor $LOF\left(x_{i}\right)$ is calculated, denoted as follows:

(5) $$LOF\left(x_{i}\right)=\frac{1}{\left|N_{k}\left(x_{i}\right)\right|}\sum_{x_{j}\in N_{k}\left(x_{i}\right)}\frac{lrd\left(x_{j}\right)}{lrd\left(x_{i}\right)}$$

$LOF\left(x_{i}\right)$ can be interpreted as the average of the ratio between the local density of data point $x_{i}$ and that of its neighbors. If $LOF\left(x_{i}\right)$ is relatively high, it indicates that $x_{i}$ is located in an area with a relatively low density and is considered an outlier that should be removed.

By utilizing the aforementioned outlier detection methods, the accuracy and robustness of outlier detection can be improved, data quality can be enhanced, and the dataset can be balanced.

The main steps in this section are shown in Algorithm 3.
**Algorithm 3.** Integrated oversampling and Outlier Detection**Input:** Feature representation dataset (with class imbalance issue)**Output:** Balanced dataset (A balanced and outlier-free synthetic dataset)**Begin** **Step 1: Integrated oversampling**   Confirm class distribution in the dataset;   Analyze the proportion of vulnerable code class and normal code class samples;   For each oversampling method in [ADASYN, SMOTE, SVMSMOTE, Borderline-SMOTE] do:   Apply the oversampling method to generate synthetic vulnerable class samples;   Store the generated synthetic samples;   End for   Merge all synthetic samples generated by different methods into a synthetic vulnerable code dataset;   Get Balanced dataset after oversampling; **Step 2: Outlier Detection**   Train One-Class SVM on the dataset to obtain the decision boundary;   For each data point *x* in the dataset do:   Evaluate *f*(*x*);   If *f*(*x*) ≥ 0 then   Mark *x* as a normal point;   Else   Mark *x* as an outlier;   End if   End for   Remove all data points marked as outliers;   For each data point in the dataset do:   Calculate the local reachability density;   Compute the LOF value;   If LOF value > threshold then   Mark the point as an outlier;   Else   Mark the point as normal;   End if   End for   Remove all data points marked as outliers;   Get Balanced dataset (a balanced and outlier-free synthetic dataset);**End**

### 3.3. Software Vulnerability Detection Based on Bi-LSTM

We will now briefly introduce the Bi-LSTM model and elaborate on its training process. Bi-LSTM (bidirectional LSTM) is a neural network model based on LSTM (long short-term memory). Traditional LSTM can only process sequential data from one direction (usually from left to right), whereas Bi-LSTM, through bidirectional learning, can capture contextual information more comprehensively. It is particularly suitable for tasks that require considering sequential dependencies (such as text comprehension in natural language processing), especially for handling tasks with complex temporal dependencies.

Zheng [18] and Lin [13] discovered in their research that there is a certain degree of correlation between AST and sentences in natural language. Hence, employing an RNN with LSTM units to capture long-term dependencies can enhance vulnerability detection performance. The Bi-LSTM neural network can learn deeper representations of potential vulnerabilities and output its prediction results. Therefore, for this paper, we selected Bi-LSTM as the training model. The neural network Bi-LSTM model structure utilized in this paper bears some similarity to that of Lin et al. [13]. The model structure is presented in Table 1.

The Bi-LSTM neural network model presented in this paper, through the combination of an embedding layer, bidirectional LSTM layer, pooling layer, and dense layer, can effectively learn and abstract information from the input sequence and employs a Dropout layer to prevent overfitting. The model ultimately utilizes a binary cross-entropy function for vulnerability prediction, with Adamax as the optimizer, ensuring efficiency and stability during the training process.

Firstly, we split the preprocessed balanced dataset into two parts, with 70% of the data used for training and 30% for testing. During the training process, the model is optimized by minimizing the loss function (in this task, the binary cross-entropy loss function). The optimizer used is Adamax, a variant of the Adam optimizer that is suitable for handling large datasets. Adamax has strong robustness during training and can effectively handle different feature scales. In this paper, we introduced an early stopping mechanism during the training process. The early stopping mechanism monitors the performance on the validation set. If the performance on the validation set does not improve over several consecutive training epochs, then training will be terminated early. This can prevent the model from overfitting on the training set and losing its generalization ability on new data. After training is completed, the performance of the model is evaluated using the test set (X_test, y_test). By evaluating the model’s performance on the test set, we can determine whether the Bi-LSTM model has sufficient detection capability to handle vulnerability detection tasks and further optimize the model based on the experimental results. To reduce the training time of the model, we implemented a multi-feature filtering and integrated sampling module. Multi-feature filtering eliminates redundant features, thereby reducing the model’s complexity and the computational resources needed for training. Simultaneously, integrated sampling optimizes data distribution, reducing the model’s reliance on extensive data and further reducing the training time. This design not only enhances the model’s training efficiency but also accelerates convergence by reducing feature dimensions and optimizing data quality.

## 4. Experimental Results and Analysis

### 4.1. Dataset

The dataset utilized in this paper is derived from the real-world dataset employed by LIN et al. [47,48], encompassing widely utilized open-source libraries such as LibTIFF, LibPNG, and Ffmpeg. These libraries have a history of security vulnerabilities. Notably, these vulnerabilities encompass types like out-of-bounds reads and buffer overflows. To construct the experimental dataset, this paper extracts vulnerability function labels from the National Vulnerability Database (NVD) and the Common Vulnerabilities and Exposures (CVE) dataset. Based on the vulnerability descriptions provided in these databases, the source code for each project’s corresponding version was downloaded, and each vulnerable function was precisely identified and annotated. The annotation rule is as follows: 1 indicates the presence of a vulnerability in the function, while 0 denotes the absence of a vulnerability.

There are 329 vulnerable function segments and 6873 non-vulnerable function segments in the dataset, with each function segment already containing a label value. The vulnerable function segments are named using CVE-IDs. Table 2 lists the datasets.

### 4.2. Experimental Environment

The CPU used in the experiment is an Intel(R) Xeon(R) Gold 6252 CPU @ 2.10 GHz, with a memory size of 38 G and a graphics card model of ASPEED Graphics Family. The Python version used in this paper is 3.11.5, and the TensorFlow version is 2.16.1. The ratio of the training set to the test set is 7:3, with 150 training epochs. The learning rate is set to 0.001 and automatically adjusted during the training process based on the training performance. Additionally, an early stopping mechanism is employed during the neural network model training process to prevent overfitting.

### 4.3. Performance Evaluation Metrics

To quantify the detection performance of MFISM, this paper employs classic classification evaluation metrics, including the accuracy, precision, recall, F1 score, Matthews correlation coefficient, and AUC-PR value. The calculation method for each metric is as follows:

Accuracy (A). The proportion of correctly classified samples to the total number of samples. The calculation method for accuracy is as follows:

(6) $$A=\frac{TP+TN}{TP+FP+TN+FN}$$

Precision (P). The proportion of correctly identified samples among all samples judged to have vulnerabilities. The calculation method for precision is as follows:

(7) $$P=\frac{TP}{TP+FP}$$

Recall rate (R). The proportion of successfully detected vulnerability samples out of all vulnerability samples. The calculation method for recall rate is as follows:

(8) $$R=\frac{TP}{FN+TP}$$

F1 score (F1). The harmonic mean of precision and recall, reflecting the overall performance of the model. The F1 calculation method is

(9) $$F1=\frac{2PR}{P+R}$$

The detection of class-balanced methods utilizes evaluation metrics such as the Matthews correlation coefficient and AUC-PR. AUC-PR: An evaluation metric used to measure the performance of classification models when dealing with imbalanced datasets. It is particularly suitable for evaluating situations where the number of positive cases is significantly smaller than the number of negative cases, such as vulnerability detection and disease diagnosis. The calculation formula is

(10) $$AUC-PR\approx \sum \left(∆Recall\times Precision\right)$$

Matthews correlation coefficient (MCC) is a metric used to assess the performance of classification models in binary classification problems. It considers the values of the confusion matrix, encompassing true positives (TPs), false positives (FPs), true negatives (TNs), and false negatives (FNs), thereby offering a balanced measure that is particularly suitable for imbalanced datasets. The formula for calculating MCC is as follows:

(11) $$MCC=\frac{\left(TP\times TN\right)-\left(FP\times FN\right)}{\sqrt{\left(TP+FP\right)\times \left(TP+FN\right)\times \left(TN+FP\right)\times \left(TN+FN\right)}}$$

In the aforementioned formula for calculating indicators, TP (true positive) denotes the number of samples where a vulnerability sample is correctly detected as a vulnerability sample, TN (true negative) denotes the number of samples where a benign sample is correctly detected as a benign sample, FP (false positive) denotes the number of samples where a vulnerability sample is incorrectly detected as a benign sample, and FN (false negative) denotes the number of samples where a benign sample is incorrectly detected as a vulnerability sample.

### 4.4. Results and Analysis

In this paper, we conducted three sets of experiments to verify the effectiveness of the proposed methods for addressing class imbalance, feature selection, and model construction.

To evaluate the model performance, in this paper, we constructed a series of experimental datasets with five different imbalance ratios, namely, IR = 10, 20, 30, 40, and 50. Taking IR = 10 as an example, we randomly selected 120 vulnerable samples and 1200 normal samples from the original data to form the vulnerable (minority) class and the normal (majority) class, respectively. Additionally, for each imbalanced dataset, we repeated the process five times to obtain five independent datasets. The calculation formula for IR is as follows:

(12) $$IR=\frac{majority\;class\;samples}{minority\;class\;samples}$$

In the context of “class imbalance”, overall classification accuracy is not a reliable metric for evaluating classifier performance. Instead, model performance is typically assessed using other commonly used learning metrics, such as Precision, Recall, and F1-score. To further validate our findings, we also incorporated two additional evaluation metrics, MCC and AUC-PR, which are particularly suitable for imbalanced datasets. In our experiments, we employed 10-fold cross-validation for each dataset to minimize the potential for bias. We applyed an ensemble-based oversampling technique specifically to the training data, rather than the entire dataset, to ensure experimental accuracy. The final results were obtained after 10 rounds of 10-fold cross-validation.

#### 4.4.1. Comparison and Analysis of Class Balancing Methods

**Figure 6:**
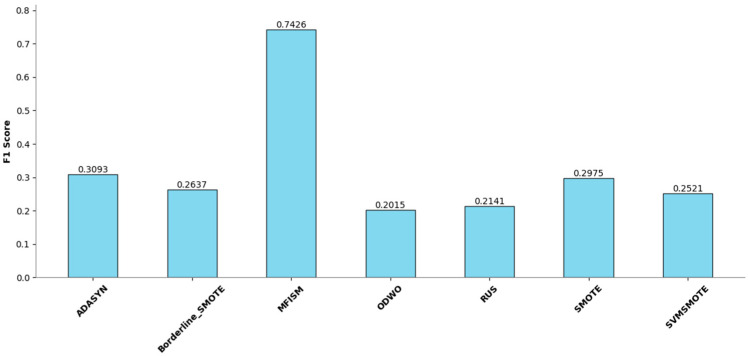
Comparison of F1 Score among various methods.

**Figure 7:**
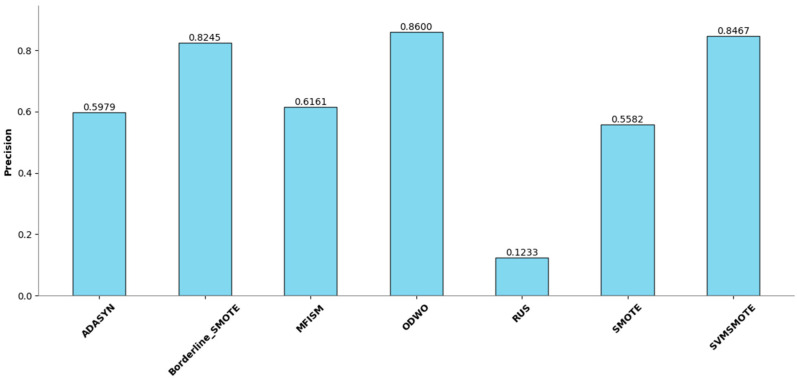
Comparison of Precision among various methods.

**Figure 8:**
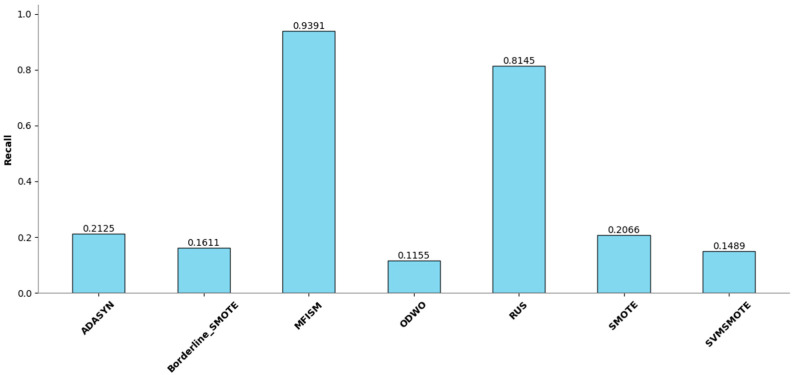
Comparison of Recall among various methods.

**Figure 9:**
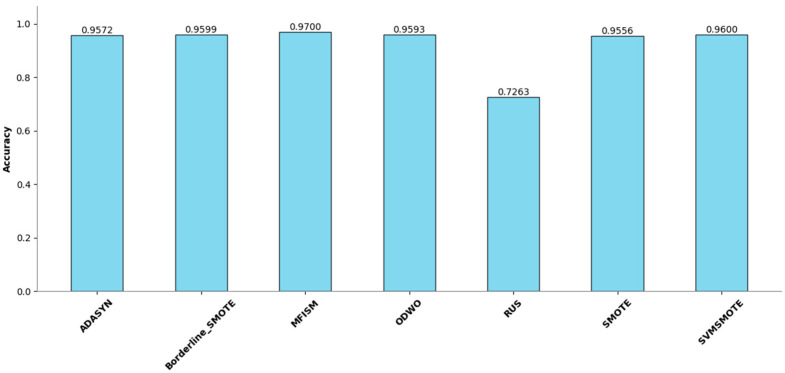
Comparison of Accuracy among various methods.

To verify the vulnerability detection performance of the class balancing method (MFISM) proposed in this paper, we conducted tests on the overall datasets of LibTIFF, LibPNG, and FFmpeg, comparing MFISM with ODWO (Original Dataset Without Oversampling) and five other advanced methods for addressing class imbalance: the ADASYN method [40], AUS method [49], SMOTE method [41], SVMSMOTE method [42], and Borderline_SMOTE method [43]. The evaluation metrics for each method are presented in Figure 6, Figure 7, Figure 8 and Figure 9.

This article evaluates the effectiveness of various oversampling methods in handling imbalanced datasets through the column chart presented in Figure 6, Figure 7, Figure 8 and Figure 9. The evaluation metrics include accuracy, precision, recall, and F1 score, utilizing a random forest model and based on tenfold cross-validation. The results reveal that MFISM excels in terms of F1 score, achieving an average of approximately 0.75, significantly surpassing other methods, notably ADASYN, which scores only about 0.40. The F1 scores for SMOTE and its variants are comparable, whereas Random Undersampling (RUS), as an undersampling method, performs poorly due to the reduction in data volume. The F1 score for the ODWO is the lowest, approximately 0.25, indicating that without oversampling, the model finds it challenging to effectively identify minority classes.

Figure 7 illustrates the performance enhancement of most oversampling methods, yet the F1 score of MFISM drops to 0.60, and ADASYN’s score experiences significant fluctuations. Although ODWO excels in accuracy, this may be attributed to the extreme imbalance in the dataset, leading the model to favor predicting the majority class. In Figure 8, MFISM leads with a recall rate of approximately 0.90, closely followed by RUS, whereas the recall rates of other methods are lower. Figure 9 reveals that, aside from RUS, the accuracy of most methods approaches 0.95, whereas RUS’s accuracy is only around 0.75.

This article effectively enhances the quality and balance of the dataset by integrating oversampling and outlier detection techniques. The experimental design employs a unified model and validation scheme, ensuring the reliability and comparability of the results. MFISM exhibits high accuracy and stability in handling imbalanced datasets, surpassing other mainstream methods. The research findings offer valuable guidance for selecting appropriate oversampling techniques and provide a more comprehensive perspective for evaluating model performance.

**Figure 10:**
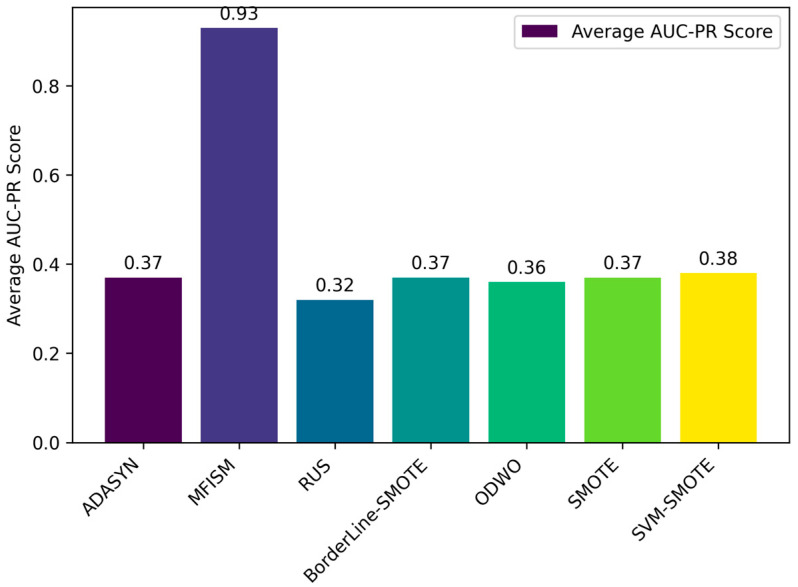
Comparison of average AUC-PR values among various methods.

**Figure 11:**
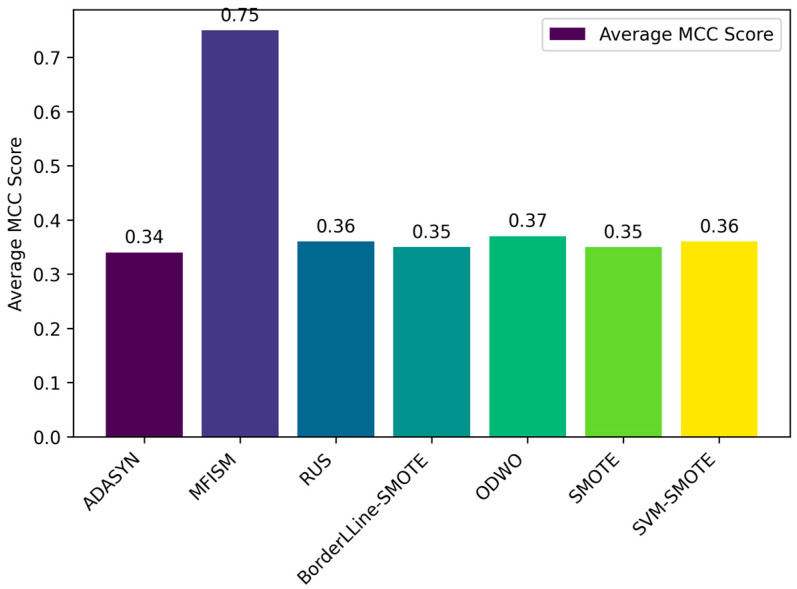
Comparison of the average MCC values among various methods.

By analyzing the original dataset, this paper further confirms the existence of significant class imbalance, where vulnerability data samples are significantly fewer than normal samples, leading to the model’s prediction results favoring the majority class samples, namely, normal data. Therefore, relying solely on accuracy and precision is insufficient to comprehensively reflect the model’s performance, especially in the case of class imbalance. To this end, this paper introduces the Matthews correlation coefficient (MCC) and the Area Under the Precision–Recall Curve (AUC-PR) as additional evaluation metrics. The closer these two metrics are to 1, the stronger the model’s predictive and discriminative abilities are. These metrics are suitable for evaluating the performance of binary classification models, as specifically demonstrated in Figure 10 and Figure 11.

Through the bar charts presented in Figure 10 and Figure 11, this paper visually demonstrates the performance of various data balancing methods for the AUC-PR and MCC metrics. The results indicate that the MFISM achieves an average score of 0.75 for MCC, surpassing traditional oversampling methods and demonstrating its exceptional performance in handling imbalanced datasets. Additionally, the MFISM attains a high score of 0.93 for AUC-PR, highlighting its robust ability to effectively identify minority class samples.

AUC-PR is a robust metric especially suitable for evaluating imbalanced datasets, as it emphasizes the identification of positive samples without overestimating model performance due to an excessive number of negative samples. This distinguishes it from AUC-ROC, which may exaggerate the model’s performance in the presence of imbalanced datasets. This paper also employs various methods to reduce data noise and enhance the quality of synthetic samples, thereby improving the accuracy and reliability of the model.

In comparison, other oversampling methods generally score below 0.4 on AUC-PR, revealing their deficiency in identifying minority class samples. Furthermore, when there is class imbalance in the dataset, relying solely on precision and accuracy may mislead the evaluation of model performance, as these metrics may not accurately reflect the model’s ability to identify minority classes.

In summary, the MFISM method excels in handling imbalanced datasets, particularly in identifying minority class samples. By adopting AUC-PR as an evaluation metric, this paper constructs a more accurate and reliable framework for assessing model performance, providing an important reference for addressing classification issues in imbalanced datasets.

#### 4.4.2. Comparison and Analysis of Feature Selection Methods

This article further screens the feature sets obtained through different methods of feature importance using ANOVA, and it can be seen that all indicators have been improved. This means that this method is effective. The DL models used are all Bi-LSTM models.

In this study, all DL models are Bi-LSTM models, DT stands for decision tree, and RF denotes random forest. By further refining the features selected by these three machine learning models, the MFISM successfully extracts feature sets that comprehensively represent vulnerability characteristics in source code. Notably, the number of selected features was not strictly limited during the feature selection process. The DT, RF, and LightGBM models all chose approximately 60 features. The experimental results indicate that most of the 60 features selected by these models are consistent, with only 20 to 30 features differing. Based on these 60 selected features, the MFISM further extracts a set of approximately 30 features. Although this results in a slight decrease in the model’s recognition performance, it demonstrates that only about 30 features are necessary to effectively describe the characteristics of source code vulnerabilities.

The data analysis presented in Table 3 clearly illustrates the performance of various feature selection methods on this dataset. The random forest model achieves an F1 score of 97.42%, slightly lower than that of the decision tree. This suggests that it has struck a good balance between precision and recall, with an accuracy rate of 97.47%. Although its overall performance is slightly inferior to the decision tree, it still maintains high classification accuracy. The decision tree model boasts an impressive F1 score of 98.01% and an accuracy rate of 98.06%, demonstrating its excellent ability to distinguish between different categories. The LightGBM model’s F1 score is 98.24% and its accuracy rate is 98.25%, indicating an optimal balance between precision and recall. Conversely, the MFISM excels in all evaluation metrics, achieving an F1 score and an accuracy rate of 99.30%, making it the top performer among all models and surpassing the other three methods. Notably, the accuracy rate of the MFISM is 1.05 percentage points higher than that of LightGBM, highlighting its advantage in enhancing classification accuracy.

Furthermore, the results demonstrate that ANOVA holds practical value in refining feature selection, aiding in the selection of the most effective feature set for the current dataset.

#### 4.4.3. Ablation Experiments

To systematically evaluate the impact of the multi-feature filtering and integrated sampling modules on model performance, we designed a series of ablation experiments based on the Bi-LSTM model. We tested four configurations:

Model 1: The baseline Bi-LSTM model without multi-feature filtering or integrated sampling.

Model 2: Bi-LSTM with multi-feature filtering but without integrated sampling.

Model 3: Bi-LSTM with integrated sampling but without multi-feature filtering.

Model 4: Bi-LSTM with both multi-feature filtering and integrated sampling.

In this study, we kept the structure and parameters of the Bi-LSTM model consistent across all four models to isolate the effects of multi-feature filtering and integrated sampling. Additionally, we introduced an early stopping mechanism during training to monitor performance metrics on the validation set and halt training when improvements plateaued. This approach helped prevent overfitting and saved training time.

The Bi-LSTM model, as the base architecture, captures both forward and backward dependencies in sequential data, making it suitable for time-series or ordered data processing. The multi-feature filtering module reduces model complexity and training time by eliminating redundant features, thereby enhancing training efficiency. The integrated sampling module optimizes data distribution, improving the model’s ability to detect minority classes and further boosting overall performance.

All models were evaluated on the same training and testing datasets to ensure fairness. We used the following metrics to assess model performance: Accuracy, Precision, Recall, F1 Score, Training Time per Epoch, and the number of epochs at early stopping. Training time and the number of epochs at early stopping were used to demonstrate the effectiveness of multi-feature filtering in reducing training time.

The results, shown in Table 4, indicate the following:

Model 1 (Baseline Bi-LSTM): Without optimization, the model achieved an accuracy of 95.13%, recall of 76.12%, F1 Score of 79.44%, and precision of 82.98%. Training stopped at epoch 22 with a total training time of 38 min.

Model 2 (Integrated Sampling): With integrated sampling, accuracy increased to 97.45%, recall to 87.92%, F1 Score to 86.77%, and precision to 85.70%. Training stopped at epoch 21 with a total training time of 92 min. The increase in training time is likely due to the computational overhead of data synthesis and adjustment.

Model 3 (Multi-Feature Filtering): With feature filtering, accuracy rose to 96.71%, recall dropped slightly to 71.88%, the F1 Score remained at 79.29%, and precision increased to 88.43%. Training stopped at epoch 19 with the total training time significantly reduced to 2 min, demonstrating a substantial reduction in model complexity and training time.

Model 4 (Multi-Feature Filtering and Integrated Sampling): Combining both modules, accuracy further improved to 97.55%, recall to 85.74%, the F1 Score to 86.55%, and precision to 87.41%. Training stopped at epoch 22 with a total training time of 6 min. This configuration not only enhanced performance but also optimized training efficiency.

These results confirm the effectiveness of each component and highlight the combined benefits of multi-feature filtering and integrated sampling in improving model performance and training efficiency.

The experimental results indicate that the integration of multi-feature filtering and integrated sampling significantly enhances the model’s performance, particularly in terms of accuracy and precision. The multi-feature filtering module reduces model complexity and training time by decreasing feature dimensions, allowing the model to achieve higher performance within fewer training rounds. The integrated sampling module addresses data imbalance by optimizing data distribution, thereby further enhancing the model’s detection capabilities for minority classes (vulnerable code). The synergistic effect of these two components yields remarkable improvements in performance, especially in terms of accuracy and recall.

As evident from Figure 3, the performance of the model has already seen some enhancement when using either multi-feature filtering (Model 3) or integrated sampling (Model 2) individually; however, the combination of the two (Model 4) achieves superior performance. Specifically, Model 2 significantly enhances accuracy, recall, and F1 scores through integrated sampling. This demonstrates that integrated sampling effectively addresses data imbalance, enabling the model to better learn the characteristics of minority categories. In software vulnerability detection, data imbalance is a common issue, with minority classes (vulnerable code) often being overlooked by the model. Integrated sampling enhances the representativeness of minority classes by synthesizing or adjusting data distribution, thereby improving the model’s vulnerability detection capabilities.

#### 4.4.4. Comparison of Classification Performance of Different DL Models

**Figure 12:**
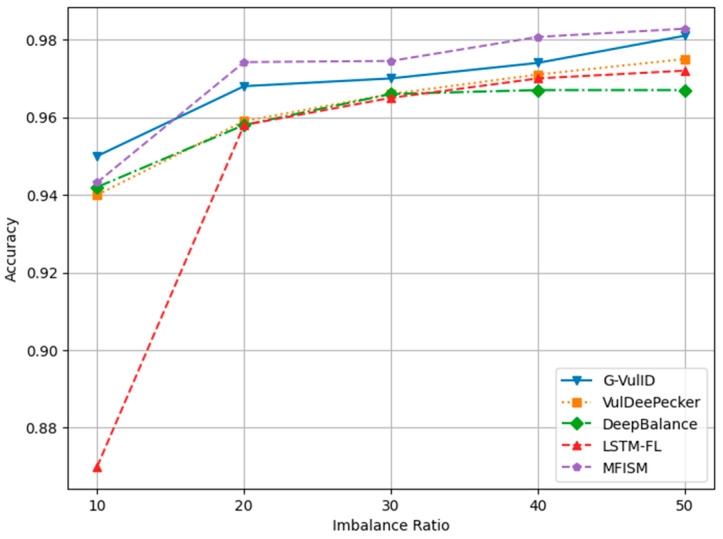
Comparison of accuracy among various models for different IR values.

**Figure 13:**
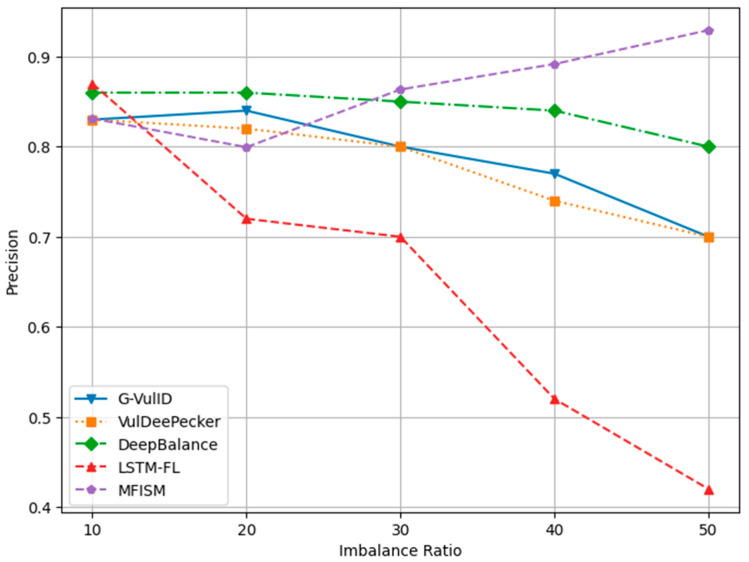
Comparison of precision among various models for different IR values.

**Figure 14:**
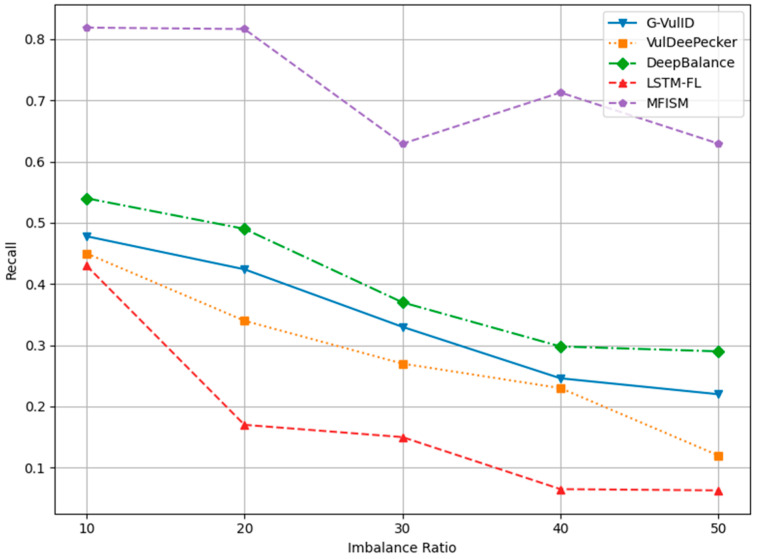
Comparison of recall among various models for different IR values.

**Figure 15:**
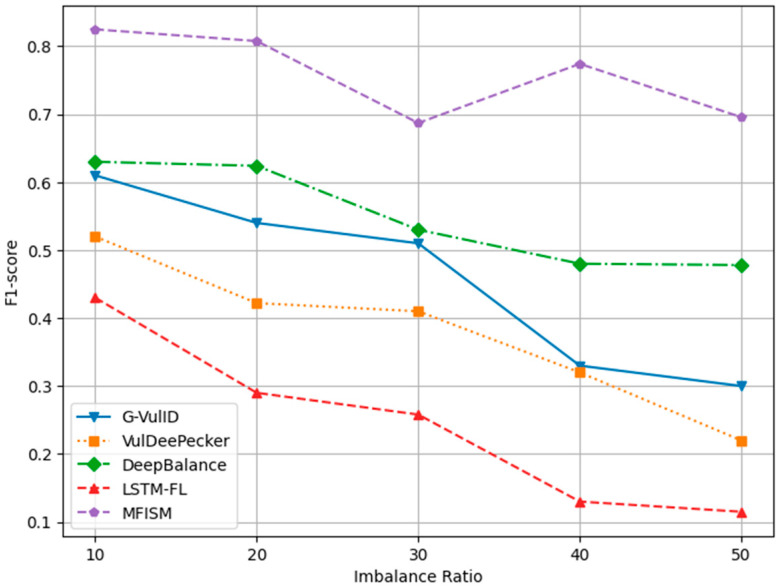
Comparison of F1-score among various models for different IR values.

To compare and verify the effectiveness of this system, five deep learning-based vulnerability detection systems similar to the research direction of this paper were selected. This paper compares the MFISM with four software vulnerability detection systems developed in the reference literature, namely, G-Vul [30], VulDeePecker [25], LSTM-FL [50], and DeepBalance [13]. These four methods were chosen because they are all detection methods based on deep learning. The DeepBalance model serves as the benchmark for this study, as its research direction closely aligns with the work presented in this paper, providing an important reference framework for our research. Figure 12, Figure 13, Figure 14 and Figure 15 display the accuracy, precision, recall rate, and F1 score of the referenced models at different imbalance rates (IRs) in broken line diagrams.

Figure 12 demonstrates that as the IR (imbalance ratio) increases, the accuracy values for all five methods exhibit a slight improvement. Figure 13 indicates that DeepBalance’s performance is slightly superior to the MFISM when the IR value ranges between 10 and 20. However, as the IR further increases, the MFISM’s performance gradually surpasses that of DeepBalance, ultimately achieving a roughly 12% improvement at the highest value. Apart from the MFISM, the accuracy of the other four methods decreases as the IR increases.

Figure 14 and Figure 15, respectively, depict the trends in recall and F1-score. Both metrics decline as the IR increases, yet the MFISM consistently outperforms. For instance, in Figure 14, the MFISM’s recall rate is generally approximately 15% higher than that of the superior DeepBalance. At IR = 10, the MFISM’s recall rate is around 0.81, but it drops to 0.62 at IR = 50. In contrast, the recall rate of the DeepBalance system, which performs best among other models at IR = 50, is merely 0.30. The experimental results reveal that, compared to other methods, the MFISM nearly excels in terms of the recall rate and F1 score. Specifically, from IR = 10 to IR = 50, the MFISM’s recall rate and F1 score values are 20% higher than those of the top-performing DeepBalance.

In addition, the experimental results indicate that VulDeePecker performs less well than the other three methods in terms of precision, recall, and F1 score indicators. This may be attributed to the classification results being biased towards the majority class when there is a significant category imbalance in the dataset, leading to a notable decrease in recall and F1 score, despite exhibiting good accuracy across different IR values. When compared to G-Vul, VulDeePecker, DeepBalance, and LSTM-FL, the MFISM gradually enhances accuracy as the IR value increases. Specifically, at an IR of 50, the MFISM’s accuracy surpasses that of the top-performing DeepBalance method by at least 11%.

VulDeePecker’s low recall rate (true-positive rate) reveals its deficiencies in identifying software vulnerabilities, particularly when addressing class imbalance issues in software vulnerability detection, as it failed to detect a significant number of functions containing vulnerabilities. According to experimental results, the MFISM achieves a notably high recall rate in identifying vulnerabilities, significantly surpassing VulDeePecker. Through effective feature selection, the MFISM outperforms the deep code characterization and seq-2-seq LSTM structure employed by DeepBalance. The experimental findings confirm that the MFISM enhances the Bi-LSTM structure’s ability to capture long-term dependencies in source code via a well-designed feature selection mechanism, which is crucial in accurately identifying potentially vulnerable code fragments. Vulnerable code frequently involves multiple interdependent statements, which are not only related to the immediate context but may also be linked to the broader code environment. The Bi-LSTM structure within the MFISM learns both forward and backward dependencies of code sequences through effective features, effectively capturing vulnerable code patterns, which is essential in identifying complex software vulnerabilities.

Figure 12, Figure 13, Figure 14 and Figure 15 demonstrate that the MFISM excels in recall rate, precision, and F1-score. A higher recall rate signifies a better true-positive rate, higher precision indicates a lower false-negative rate, and a higher F1-score reflects the superior overall performance of the MFISM. Consequently, the MFISM can detect more vulnerabilities with a lower false-positive rate and a higher true-positive rate, highlighting its advantages in addressing class imbalance issues in software vulnerability detection.

In this study, to comprehensively evaluate the performance of the proposed MFISM in function-level vulnerability detection, we selected a range of classic and state-of-the-art algorithmic models for comparison. These models include Bi-LSTM, TextCNN [51], RoBERTa [52], CodeBERT [28], Devign [53], and ReGVD [20]. Spanning from traditional recurrent neural networks to the latest pre-trained language models and graph neural network approaches, these models provide a robust benchmark for our research.

We utilized the real benchmark dataset from CodeXGLUE [54] for function-level vulnerability detection. This dataset, created by Zhou et al. [53], comprises 27,318 manually labeled vulnerable and non-vulnerable functions extracted from two large C-language open-source projects, QEMU and FFmpeg. Given the balanced nature of the dataset in terms of positive and negative samples, we conducted experiments by introducing the MFISM solely based on the Bi-LSTM model described in Section 3.3. The data preprocessing and multi-feature filtering stages remained consistent with the previous sections. The experimental results are shown in Table 5, displaying the accuracy of different models on the test set.

In Table 5, Idx and UniT denote the index-focused graph structure and the unique token-focused graph structure, respectively. CB and G-CB represent the use of token embeddings from CodeBERT and GraphCodeBERT [55] alone to initialize node features.

As shown in Table 5, the MFISM achieves significant performance improvements over models such as Bi-LSTM, TextCNN, RoBERTa, and CodeBERT when only the multi-feature filtering module is introduced, reaching an accuracy of 66.41%, which is higher than ReGVD’s 63.69%. This result indicates that the MFISM, through its multi-feature filtering module, can efficiently focus on features significantly related to vulnerability detection while removing redundant features and reducing model complexity.

Moreover, the MFISM demonstrates a notable advantage in training time. This is attributed to the multi-feature filtering module, which reduces feature dimensions and optimizes training efficiency. In the experiments, the MFISM not only achieves accuracy close to ReGVD but also outperforms other baseline models in training time, making it more feasible for practical applications. Specifically, the MFISM achieves an accuracy of 66.41% on the CodeXGLUE vulnerability detection dataset, proving its effectiveness in vulnerability detection tasks. Compared to Devign and ReGVD, the MFISM can achieve near-optimal results without relying on complex graph structures or pre-trained models. This shows that the MFISM, through its multi-feature filtering module, can effectively capture key features in the code, enabling efficient vulnerability detection.

In summary, the MFISM performs well in vulnerability detection tasks through its multi-feature filtering module, achieving a performance close to state-of-the-art benchmark models while significantly improving training efficiency. These results demonstrate that the MFISM is an efficient and effective vulnerability detection method, providing strong support for vulnerability detection in practical applications. Experimental results reveal that the MFISM’s key evaluation metrics surpass those of other methods across various IR values, affirming the superiority of our feature selection over the deep code features extracted by deep learning models. Furthermore, our experiments showed that the DeepBalance model, which exhibited the best performance, required a training time of 6 h and 10 min, whereas the MFISM reduced the training time to approximately 2 h through the implementation of our feature selection approach. This substantial improvement in time efficiency, especially in the context of enhancing overall performance, indirectly validates the efficiency of the MFISM method. Refer to Table 6 for details.

The MFISM effectively addresses the issue of category imbalance and generates more diverse datasets by integrating sampling learning into various oversampling techniques. Additionally, the use of outlier detection technology further enhances data quality, thereby improving the model’s ability to recognize minority class samples and significantly boosting overall performance.

To achieve these improvements, this paper employs multi-dimensional optimization to significantly reduce the model training time. The core of this optimization lies in decreasing feature dimensions, optimizing model complexity, enhancing training efficiency, and reducing the risk of overfitting through scientific feature selection strategies. Specifically, ANOVA is used to screen features significantly related to the target variable, effectively eliminating redundant or irrelevant features and thus significantly reducing feature dimensions. This process not only decreases the computational load during model training but also avoids the “curse of dimensionality”, where the number of parameters that the model needs to handle grows exponentially in high-dimensional space, leading to low training efficiency.

After feature selection, the model structure becomes more concise, and the complexity is optimized, significantly reducing the number of parameters to be processed during training. This not only substantially decreases the training time but also facilitates convergence. Additionally, the filtered feature set is more representative and can support model learning more efficiently, enabling the model to achieve better performance with fewer training iterations, thereby further shortening the training time. Simultaneously, removing irrelevant features enhances the model’s generalization ability, significantly reduces the risk of overfitting, improves the stability and adaptability of the model, and further accelerates the convergence speed.

In summary, the MFISM significantly reduces the model training time and enhances the model’s generalization ability and stability by reducing feature dimensions, optimizing model complexity, enhancing training efficiency, and reducing the risk of overfitting. This approach provides strong support for efficient model development in practical applications.

By integrating these optimizations, the Bi-LSTM neural network in the MFISM can more precisely discern the feature differences between vulnerable and normal code. The preliminary feature importance screening, coupled with ANOVA, refines a set of key features. This not only enhances the vulnerability detection capabilities of the Bi-LSTM neural network but also significantly reduces the training time.

## 5. Summary

To enhance the detection performance of software vulnerability detection models and address the limitations of existing methods, this paper introduces a novel model, MFISM-Bi-LSTM. This model tackles the issues of lengthy training times and limited dataset sizes by incorporating multi-feature filtering and integrated oversampling techniques. It extracts features from source code and balances feature data, providing crucial feature representations for learning vulnerability characteristics. This approach significantly boosts the accuracy of the Bi-LSTM model in detecting source code vulnerabilities.

The effectiveness of the proposed model is validated through extensive evaluation on datasets from three real-world project source codes. The results indicate that the MFISM-Bi-LSTM outperforms the DeepBalance model in terms of F1 scores, with a performance improvement of 10%. Additionally, the training time for the Bi-LSTM model is reduced to 2–3 h, underscoring its potential for source code vulnerability detection.

Reducing model training time is crucial for enhancing the feasibility and efficiency of the model in practical applications. Multi-feature filtering reduces model complexity by eliminating redundant and irrelevant features. This process decreases the number of parameters that the model needs to process during training and selects features significantly related to vulnerability detection through feature importance assessment and ANOVA analysis. This enables the model to learn key features more efficiently, reducing computational resources and the training time.

Integrated sampling addresses data imbalance by synthesizing or adjusting data distribution, enhancing the model’s ability to detect minority classes (vulnerable code). By synthesizing minority class samples through various oversampling methods and removing noisy samples via outlier detection, data quality is improved. This high-quality dataset allows the model to converge faster during training, reducing the number of training epochs.

The combination of multi-feature filtering and integrated sampling not only boosts model performance but also significantly reduces the training time. Multi-feature filtering lowers model complexity and computational demands, while integrated sampling optimizes data distribution, reducing the model’s reliance on large datasets. This synergy enables the model to achieve higher performance in fewer training epochs.

In practical applications, reducing the training time enhances model development efficiency, allowing developers to adjust model parameters and conduct experimental validations more quickly. This accelerates model development and deployment. The reduced training time also means less dependence on computational resources, lowering operational costs and making the model more suitable for resource-constrained environments. Additionally, by reducing feature dimensions and optimizing data distribution, the model not only trains faster but also improves its generalization ability, demonstrating higher robustness when faced with new, unseen data.

In summary, through the synergistic effects of multi-feature filtering and integrated sampling, we have significantly enhanced model performance and drastically reduced the training time, providing strong support for efficient model development in practical applications.

Nonetheless, the model’s application efficacy necessitates further refinement. Presently, the exploration is confined to the intermediate representation of the AST, implying that the MFISM still has room for enhancement. Future efforts will focus on investigating additional graph structure representations to expand the model’s applicability.

Moreover, the current limitation to C/C++ structure types and the rudimentary classification-based vulnerability detection model restrict the detection of diverse application vulnerabilities across multiple programming languages. Future work will prioritize in-depth analyses of multi-classification vulnerability detection models, collaborative exploration with other vulnerability detection models across various programming languages, and a reduction in detection granularity to develop more precise detection methodologies.

## Figures and Tables

**Table 1:** Bi-LSTM model structure.

Layer	Activation Function	Output Shape	Param
Embedding	None	(batch_size,30,100)	1,025,000
Bi-LSTM	‘tanh’	(batch_size,30,128)	84,480
GolbalMaxPooling	None	(batch_size,128)	0
Dense1	‘tanh’	(batch_size,128)	16,512
Dense2	None	(batch_size,64)	8256
Dense3	‘sigmoid’	(batch_size,1)	65

**Table 2:** Dataset information.

Dataset	Func_Total	Non_Func	Vul_Func
LibTIFF	5757	5565	192
LibPNG	621	577	44
FFmpeg	825	731	94
Final_data	7202	6873	329

**Table 3:** Comparison of feature selection methods.

Feature_Method	Precision	Recall	F1_Score	Accuracy
DL_DT	98.12%	97.92%	98.01%	98.06%
DL_RF	97.53%	97.65%	97.42%	97.47%
DL_lightGBM	98.26%	98.18%	98.24%	98.25%
MFISM	99.29%	99.28%	99.30%	99.30%

**Table 4:** Performance metrics and training efficiency of different model configurations.

Model	Precision	Recall	F1_Score	Accuracy	Training Time per Epoch
Model1	82.98%	76.12%	79.44%	95.13%	38 m/22 epochs
Model2	85.70%	87.92%	86.77%	97.45%	92 m/21 epochs
Model3	88.43%	71.88%	79.29%	96.71%	2 m/19 epochs
Model4	87.41%	85.74%	86.55%	97.55%	6 m/22 epochs

**Table 5:** Accuracy of vulnerability detection on the test set.

Model	Accuracy
Bi-LSTM	59.37%
TextCNN	60.69%
RoBERTa	61.05%
CodeBERT	62.08%
Devign (Idx + CB)	60.43%
ReGVD (GCN + UniT + G-CB)	63.69%
MFISM	66.41%

**Table 6:** Comparison of training time between the MFISM and DeepBalance.

Model	Training Duration
DeepBalance	6 h 10 min
MFISM	2 h 18 min

## Data Availability

The datasets used in this article were publicly released by Lin [47,48] and Zhou [53] and are available at the following URLs: https://github.com/DanielLin1986/function_representation_learning (accessed on 24 January 2025), https://github.com/DanielLin1986/TransferRepresentationLearning (accessed on 24 January 2025), and https://github.com/daiquocnguyen/GNN-ReGVD (accessed on 24 January 2025).

## Abbreviations

The following abbreviations are used in this manuscript:

MFISM	multi-feature screening and integrated sampling model
AST	abstract syntax tree
ANOVA	analysis of variance
DL	deep learning
ML	machine learning
CNNs	convolutional neural networks
RNN	recurrent neural network
BRNN	bidirectional recurrent neural network
LSTM	long short-term memory network
Bi-LSTM	bidirectional long short-term memory network
NVD	National Vulnerability Database
SARD	Software Assurance Reference Dataset
CVE	Common Vulnerabilities and Exposures dataset
CFG	control flow graph
PDG	program dependency graph
IOS	integrated oversampling
LOF	local outlier factor
ODWO	Original Data Without Oversampling
ADASYN	Adaptive Synthetic Sampling
RUS	random undersampling
SMOTE	Synthetic Minority Oversampling Technique
MCC	Matthews correlation coefficient
AUC-PR	Area Under the Precision–Recall Curve
IR	imbalance ratio
BGNN4VD	Bidirectional Graph Neural Network for Vulnerability Detection
